# Effects of Epicatechin on the Expression of MyomiRs−31, −133, −136, −206, −296, and −486 in the Skeletal Muscle of the Offspring of Obese Mothers

**DOI:** 10.1007/s12013-025-01700-x

**Published:** 2025-02-27

**Authors:** Paola B. Zárate-Segura, Ana Luisa Alvarez-Chávez, Sergio De los Santos, Fernando G. Bastida-Gonzalez, José Manuel Hernández-Hernández, Elena Zambrano, Ramón Mauricio Coral-Vázquez, Patricia Canto

**Affiliations:** 1 Laboratorio de Medicina Traslacional, Escuela Superior de Medicina, Instituto Politécnico Nacional, Ciudad de México, México; 2 Unidad de Investigación en Obesidad, Facultad de Medicina, Universidad Nacional Autónoma de México, Ciudad de México, México; 3 Subdirección de Investigación Clínica, Dirección de Investigación, Instituto Nacional de Ciencias Médicas y Nutrición “Salvador Zubirán”, Ciudad de México, México; 4 Laboratorio Estatal de Salud Pública del Estado de México, ISEM, Estado de México, México; 5 Departamento de Genética y Biología Molecular, Centro de Investigación y de Estudios Avanzados, CINVESTAV-IPN, Ciudad de México, México; 6 Departamento de Biología de Reproducción, Instituto Nacional de Ciencias Médicas y Nutrición “Salvador Zubirán”, Ciudad de México, México; 7 Sección de Estudios de Posgrado e Investigación, Escuela Superior de Medicina, Instituto Politécnico Nacional, Ciudad de México, México; 8 Subdirección de Enseñanza e Investigación, Centro Médico Nacional “20 de Noviembre”, Instituto de Seguridad y Servicios Sociales de los Trabajadores del Estado, Ciudad de México, México

**Keywords:** (-)-Epicatechin, Obese by programming, Gastrocnemius muscle, Soleus muscle, myomiRNAs.

## Abstract

**Specific myogenic microRNAs termed “myomiRNAs”** are involved in skeletal muscle development and regeneration, and an obesogenic environment *in utero* may affect these processes. The present study aimed to determine whether this environment induced variations in the expression levels of myomiRs-31, −133, −136, −206, and −296 and whether the administration of (-)-epicatechin (Epi), an exercise mimetic, could modify these variations. Rat Wistar male offspring from control mothers (C) or obese mothers (MO) were treated (C+Epi and MO+Epi) or not treated with Epi (C and MO). MyomiRNA expression in the gastrocnemius and soleus muscles was analyzed via RT‒qPCR, and bioinformatic analysis was used to predict the participation of these miRNAs in different skeletal muscle signal transduction pathways. The expression of myomiRNA-31-5p in the gastrocnemius and soleus was significantly lower in the Epi-treated groups (C+Epi and MO+Epi vs. C and MO). The expression of myomiRNA-206 increased in the gastrocnemius muscles of the MO and MO+Epi groups but decreased in the soleus muscles of the MO and MO+Epi groups. The expression of myomiRNA-296 was increased in the MO group in the gastrocnemius and soleus but was reduced in the Epi stimulus group. The expression of myomiRNA-486 increased in the gastrocnemius of the C+Epi group and decreased in the soleus of the MO+Epi group (*p* = 0.028 vs. MO). In conclusion, we show that an intrauterine obesogenic environment differentially affects the expression levels of some myomiRNAs and that this effect is modified by epicatechin.

## Introduction

The exposure of fetuses to an obesogenic environment *in utero* leads to an increased risk of developing several diseases in the early adulthood of the progeny (fetal programming) [1–4]. This is important, as the prevalence of obesity in women of reproductive age has increased [4, 5]; therefore, a significant proportion of women planning to become pregnant have obesity.

The embryonic development of animal skeletal muscle is precisely orchestrated [6]. This process can be affected by various stimulus that can impact several signaling pathways, activating specific transcription factors and reprogramming gene expression [7]. For this reason, an unfavorable maternal environment, such as an obesogenic stimulus, could have deleterious impacts on the offspring of obese mothers by reducing the muscle cross-sectional area and the number of fibers, as well as a reduction in the proliferation of muscle satellite cells and myogenic markers postnatally [8–10].

In this context, microRNAs (miRNAs), a class of noncoding RNAs, play essential roles in regulating several biological processes. A type of miRNA called a myomiRNA is predominantly expressed in skeletal muscle tissue and plays a role in the development, regulation, and function of the muscle, as well as the homeostasis of the skeletal muscle [11, 12]. In addition, many nonmuscle-specific miRNAs are also required for cell proliferation and skeletal muscle differentiation [10, 13].

Additionally, (-)-epicatechin (Epi), a flavonoid present in large quantities in cocoa (*Theobroma cacao*), tea (*Camellia sinensis*) and grape (*Vitis vinifera*) [14], has beneficial effects on skeletal muscle since it decreases fibrosis, improves its function [15], induces muscle mitochondrial biogenesis [16] and enhances the repair process of this tissue [17]. Furthermore, in vivo studies in an obesity model revealed that Epi inhibited obesity-associated skeletal muscle loss [18]. In a programmed obesity model, Epi administration increased lean mass [9].

On the other hand, some miRNAs are essential for muscle function (miRNA-486) [19], participate in muscle myogenesis (miRNA-136) [20], promote muscle regeneration (miRNA 206) [21], and are upregulated (miRNA-133) or suppress myogenesis (miRNA-31-5p) [22]. Other miRNAs, such as miRNA-296, are related to obesity and type 2 diabetes [23].

A previous study by our research group revealed that the offspring of mothers obese have a significant increase in the amount of total and visceral fat; as well as a decreased lean mass [9]. However, to our knowledge, no studies have focused on whether the obesogenic environment [10, 11] can modify the expression of myomiRNAs in offspring. Therefore, our study aimed to determine whether obese rat offspring show, by programming, a modification in the expression levels of myomiRs-31-5p, −133, −136, −206, −296-3p, and −486 determined by qRT‒PCR in the gastrocnemius and soleus muscles and whether the administration of Epi, an exercise mimetic [24], could modify these effects.

## Materials and Methods

### Animal Model

Wistar rat protocols were approved by the Ethics Committee in Animal Experimentation of the Salvador Zubiran National Institute of Medical Sciences, reference CINVA: UIO-1892-17/19-1, and by the Research and Ethics Commissions of the Faculty of Medicine, National Autonomous University of Mexico, reference FM/DI/105/2019. In addition, all of the animal experiments complied with the ARRIVE guidelines and followed the U.K. Animals (Scientific Procedures) Act, 1986 and associated guidelines, as well as the Guidelines for the Care and Use of Laboratory Animals from the Laboratory Animal Resources Institute (http://www.nal.usda.gov/awic/animal-welfare-act), which were applied following the Mexican Official Norma NOM-062-ZOO-1999.

The experimental procedures used to induce maternal obesity (F0) to obtain the F1 offspring and the intervention with (-)-epicatechin were previously described in detail by our research group [9]. Briefly, at weaning, sixteen female albino Wistar rats (F0) from different litters were randomly assigned to the control group (C, *n* = 8), which was fed a laboratory chow diet (energy of 4.07 kcal/g), or the maternal obesity group (MO, *n* = 8), which was fed a high-fat diet (HFD) (energy of 4.9 kcal/g). At postnatal day (pnd) 120, all F0 female albino rats were placed with sexually active male breeders (fed a chow diet all the time) and conceived during the next cycle. Both groups (C and MO) were fed their respective diets (chow or HFD) during pregnancy and lactation.

The F1 pups were weaned at postnatal day 21, all received a chow diet from weaning to euthanasia, and only males were analyzed. For the (-)-epicatechin protocol, the pups received vehicle (water) or 1 mg/kg body mass each 12 h of Epi treatment via an oral catheter (Sigma‒Aldrich, St. Louis, MO, U.S.A.) [15, 25, 26] from pnd 21–110 (13 weeks) [27]. Eight male descendants from the control or maternal obesity groups from different litters were randomly selected for the control groups (vehicle: C and MO) or, the (-)-epicatechin intervention groups (C+Epi or MO+Epi) (Supplementary Fig. 1). No adverse effects were observed during this dietary intervention.

### Gastrocnemius and Soleus Tissue RNA Extraction

At 110 postnatal days, the male pups were sacrificed after being anesthetized with an isoflurane inhaler, and the gastrocnemius and soleus were obtained. RNA extraction from both skeletal muscle tissue samples was performed with Invitrogen® TRIzol reagent (Invitrogen, U.S.A.), according to the instructions provided by the manufacturer. Briefly, the RNA obtained was treated at room temperature for 15 min with 10U of DNase 1 (Roche, Indianapolis, IN, USA), and the nucleases were inactivated at 80 °C for 20 min. RNA was precipitated at −20 °C for 20 min after adding 2.5 volumes of ethanol and 1/10 volumes of sodium acetate 3 M. Finally, RNA concentration and purity were assessed by U.V. spectrophotometry with Nanodrop ND-1000 (Thermo Fisher Scientific, Waltham, MA). After RNA isolation, all samples were immediately frozen and stored at −80 °C.

### Reverse Transcription‒Quantitative Polymerase Chain Reaction (RT‒qPCR) Analyses of MiRNAs

Details of the RT‒qPCR analyses were described previously [28]. The TaqMan Advanced MicroRNA Assays from Applied Biosystems used in this study were as follows: assay ID: 000185 for mmu-miR-31-5p; assay ID: 001637 for mmu-miR-133a-5p; assay ID: 002511 for mmu-miR-136-5p; assay ID: 000510 for mmu-miR-206-3p; assay ID: 002101 for mmu-miR-296-3p; and assay ID: 002093 for mmu-miR-486. All samples were normalized to U6 RNA expression (internal control), and fold changes were calculated through relative quantification.

### MyomiRNAs Bioinformatics Analysis

Skeletal muscle-specific expression genes and pathways were downloaded from the NeuroMuscle DB (http://yu-mbl-muscledb.com/NeuroMuscleDB/), an extensive gene database on muscle development and neuromuscular disorders. MirDIP was used for the bidirectional analysis of myomiRNA targets expressed in SkM with at least three positive prediction filters [29]. To determine the routes and biological processes and predict interactions in which the myomiRNAs regulate the targets, including the miRNAs studied experimentally, we used ShinyGO v0. 75 [30].

### Statistical Analysis

The data were analyzed with GraphPad Prism 6.0 software (GraphPad Software, San Diego, CA) and tested for a normal distribution using the D’Agostino & Pearson test for all groups. The results are expressed as the mean±standard deviation for data that were normally distributed evaluated by the Saphiro-Wilk test and as the median ± range for data that were not normally distributed across eight individual experimental observations. Two-way ANOVA was performed for normally distributed data to evaluate the interaction effects of the maternal diet and Epi treatment. In addition to the evaluation of the interaction between the two variables, the overall contribution of each variable to the variation of the model was evaluated with the two-way ANOVA, which compared the two groups of descendants from control mothers (C and C+Epi) with the two groups of descendants from obese mothers (MO and MO+Epi) to evaluate the contribution of the maternal diet to the model. The same was assessed with the Epi treatment, comparing the two groups without Epi administration (C and MO) vs. the two groups with Epi treatment for 90 days (C+Epi and Mo+Epi), to determine the contribution of the Epi treatment to the variation in the model. Significant differences were defined by *p* < 0.05. We evaluated the homogeneity of variances of the data sets with the Brown-Forsythe test, in the case in which this criterion was met, we performed a Tukey *post hoc* test for multiples comparisons, if not, we performed a Bonferroni test for the multiple’s comparisons evaluation. The statistical results of the assumption checks including normality and homogeneity of variance of the different myomiRs in gastrocnemius and soleus muscles are present in the Supplementary Table 1.

Significant differences were defined by a *p* < 0.05. When the normality assumption was not met, a Kruskal‒Wallis nonparametric test was performed with Dunn’s post hoc test for multiple comparisons. A value of *p* < 0.05 was considered statistically significant.

## Results

**Fig. 1:**
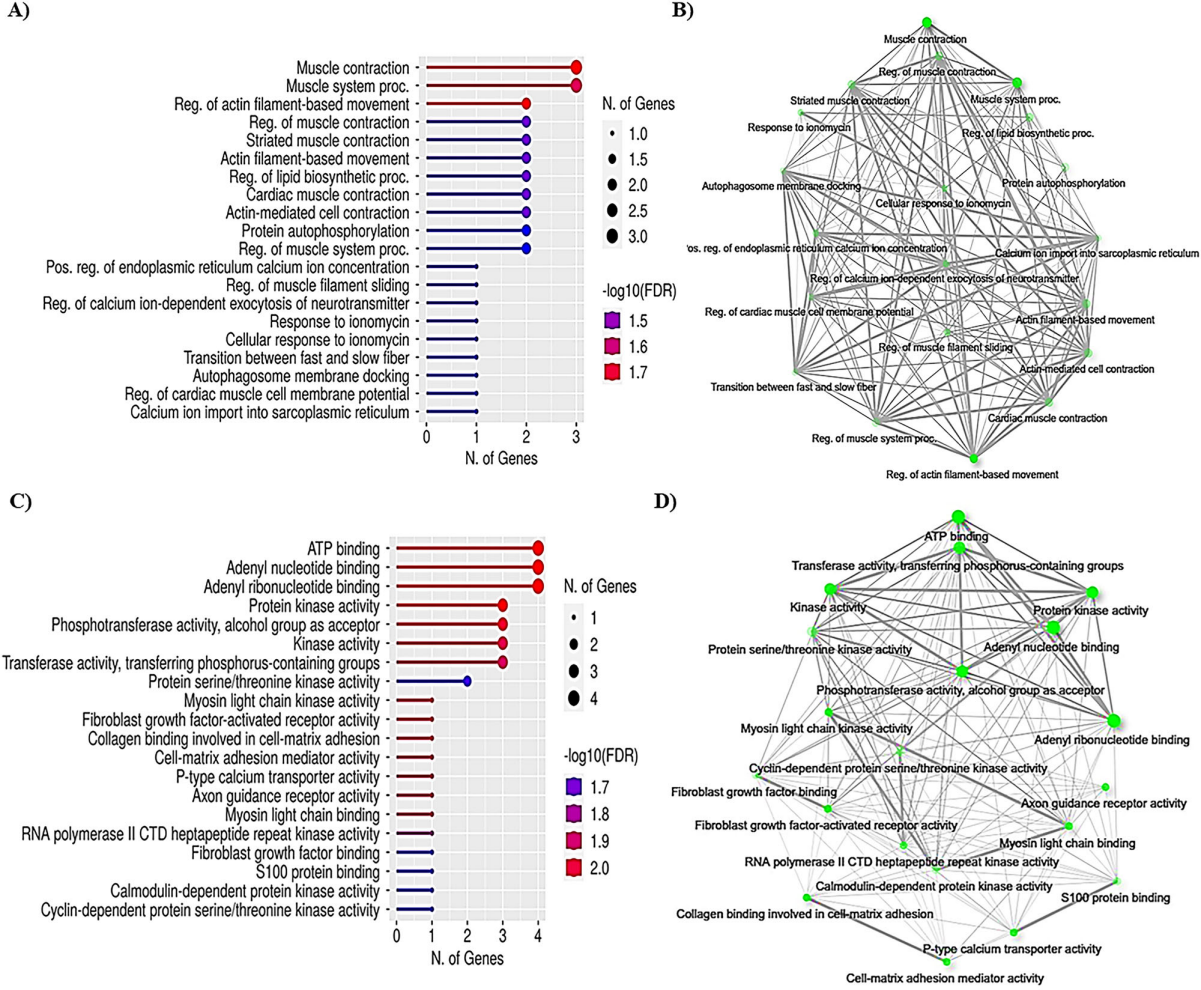
MyomiRNA−31, −133, −136, −206, −296, and −486 regulate skeletal muscle genes. Ontology of gene biological processes in which the studied myomiRNA regulate skeletal muscle functions **A**) and interaction graphs of these biological processes **B**). Ontology of molecular process genes regulated in skeletal muscle by the studied myomiRNAs **C**) and interaction graphs of these molecular processes **D**). Analysis was carried out with http://ophid.utoronto.ca/mirDIP/index_confirm.jsp#r and http://bioinformatics.sdstate.edu/go/

To explore the participation of the different miRNAs in the biological and molecular processes of skeletal muscle, we carried out an initial bioinformatic analysis. Fig. 1 shows the ontologies of the biological processes (Fig. 1A, B) and molecular functions (Fig. 1C, D) that could be regulated in skeletal muscle by the myomiRNAs −31-5p, −133, −136, −206, −296, and −486. Interestingly, most regulated biological pathways are involved in muscle contraction and hypertrophy. With respect to molecular function, the signal transduction pathways with the most genes involved were ATP binding, adenyl nucleotide binding, and cytoskeletal protein binding. Fig. 1B, D show the interaction networks of biological processes and molecular functions that could be regulated by myomiRNAs-31-5p, −133, −136, −206, −296, and −486.

Since Epi modifies the expression of myomiRNAs that are activated during physical exercise [28], we evaluated the effect of this flavonol on the expression levels of myomiRNAs-31-5p, −133, −136, −206, −296, and −486 in the gastrocnemius and soleus of eight offspring of obese mothers. Two-way ANOVA was performed after the data were tested for a normal distribution.

**Fig. 2:**
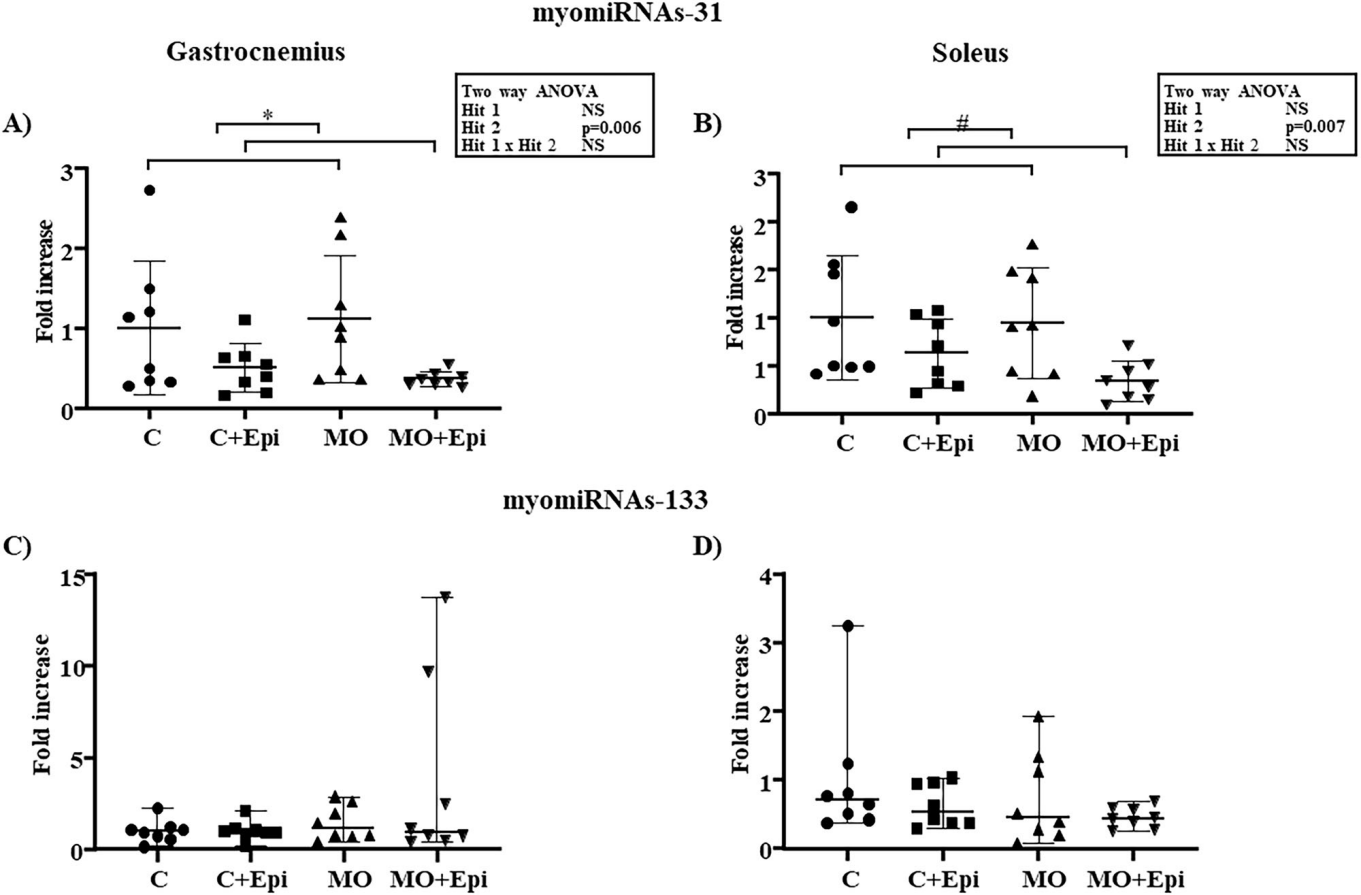
Relative myomiRNA−31 and −133 expression in the gastrocnemius and soleus muscles of the Epi-treated offspring of obese mothers. Offspring of the control group or obese mothers (MOs) were treated with Epi for 13 weeks. RT‒qPCR was used to analyze miR-31 expression, and the data were analyzed via two-way ANOVA (maternal diet × postnatal Epi treatment). For myomiRNA-31, postnatal Epi treatment induced significant decreases in expression in the gastrocnemius (**Panel A**, F (1,28) = 8.689, 23.45% variation *p = 0.006) and soleus (**Panel B**, F (1,28) = 8.355, 22.06% variation, ^#^*p* = 0.007) muscles. Hit 1 indicates C and C+Epi vs. MO and MO+Epi; Hit 2 indicates C and MO vs. C+Epi and MO+Epi. There was no interaction between the two stimuli. The results were also analyzed with a post hoc Tukey test for paired comparisons if the variance homogeneity criteria were met or with a Bonferroni post hoc correction if it not. This analysis revealed no significant differences. For myomiRNA-133, the data are expressed as medians and ranges and were analyzed via a Kruskal‒Wallis nonparametric test followed by a post hoc Dunn test for pairwise comparisons. No significant differences were observed in the gastrocnemius (**Panel C**) or soleus (**Panel D**) muscles. NS not significant. *n* = 8 rats per group. Epi (-)-epicatechin, C control offspring, MO maternal obesity offspring, C+Epi control offspring treated with Epi, MO+Epi maternal obesity offspring treated with Epi

After the data were tested for a normal distribution, two-way ANOVA was performed on myomiRNA-31-5p data. Although we did not find any interaction effect between the maternal diet and the Epi treatment or a main effect of the maternal diet on miRNA expression, we detected a significant reduction in the expression of both the gastrocnemius (Fig. 2 Panel A, *p* = 0.006) and soleus muscles (Fig. 2 Panel B, *p* = 0.007), accounting for 23.45% and 22.06%, respectively, of the model variation due to the administration of Epi. When we compared the groups with a one-way ANOVA, we did not find any significant differences.

**Fig. 3:**
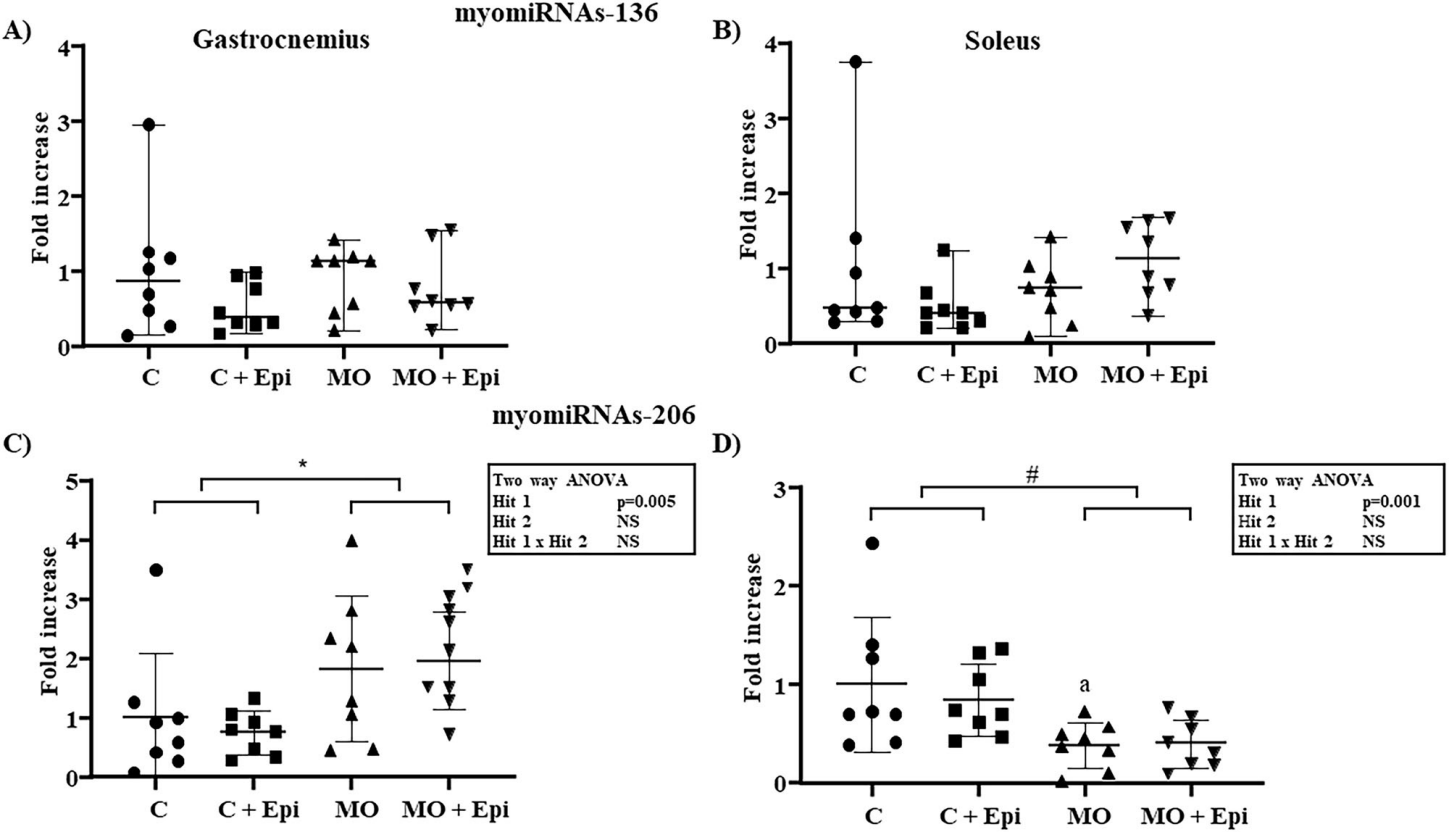
Relative myomiRNA−136 and −206 expression in the gastrocnemius and soleus muscles of the Epi-treated offspring of obese mothers. RT‒qPCR analysis of myomiRNA**-**136 revealed no significant difference between the gastrocnemius (**Panel A**) and soleus (**Panel B**) groups. Data expressed as medians and ranges were analyzed using the Kruskal‒Wallis nonparametric test and a post hoc Dunn test for pairwise comparisons. For myomiRNA-206, the data are expressed as the means and standard deviations and were analyzed using two-way ANOVA (maternal diet × postnatal Epi treatment). Maternal obesity induced an increase in the expression of the myomiRNAs in the gastrocnemius (**Panel C**) (C and C+Epi vs. MO and MO+Epi; F (1,28) = 9.382, 24.85% variation, *p = 0.005). In contrast, in the soleus (**Panel D**), maternal obesity induced a reduction in the expression of the myomiRNAs (C and C+Epi vs. MO and MO+Epi; F (1,28) = 12.49, 30.40% variation, ^#^*p* = 0.001). There was no interaction between the two stimuli. A post hoc Tukey test for pairwise comparisons was also used to analyze the data. ^a^*p* = 0.033 vs. C. NS not significant. *n* = 8 rats per group. Epi (-)-epicatechin, C control offspring, MO maternal obesity offspring, C+Epi control offspring treated with Epi, MO+Epi maternal obesity offspring treated with Epi

In addition, there was no statistically significant difference between the groups for myomiRNA-133 (Fig. 2 Panel C, D) or myomiRNA-136 (Fig. 3 Panel A, B).

The myomiRNA-206 data met the normality criteria, so two-way ANOVA was performed. An overall effect of the maternal diet on the model was evident when the two groups of descendants of control mothers (C and C+Epi) were compared with the groups of descendants of obese mothers (MO and MO+Epi). In the gastrocnemius, a significant increase in the expression of the miRNA was evident (Fig. 3 Panel C, *p* = 0.005); in contrast, in the soleus, a significant reduction was detected (Fig. 3 Panel D, *p* = 0.001).

**Fig. 4:**
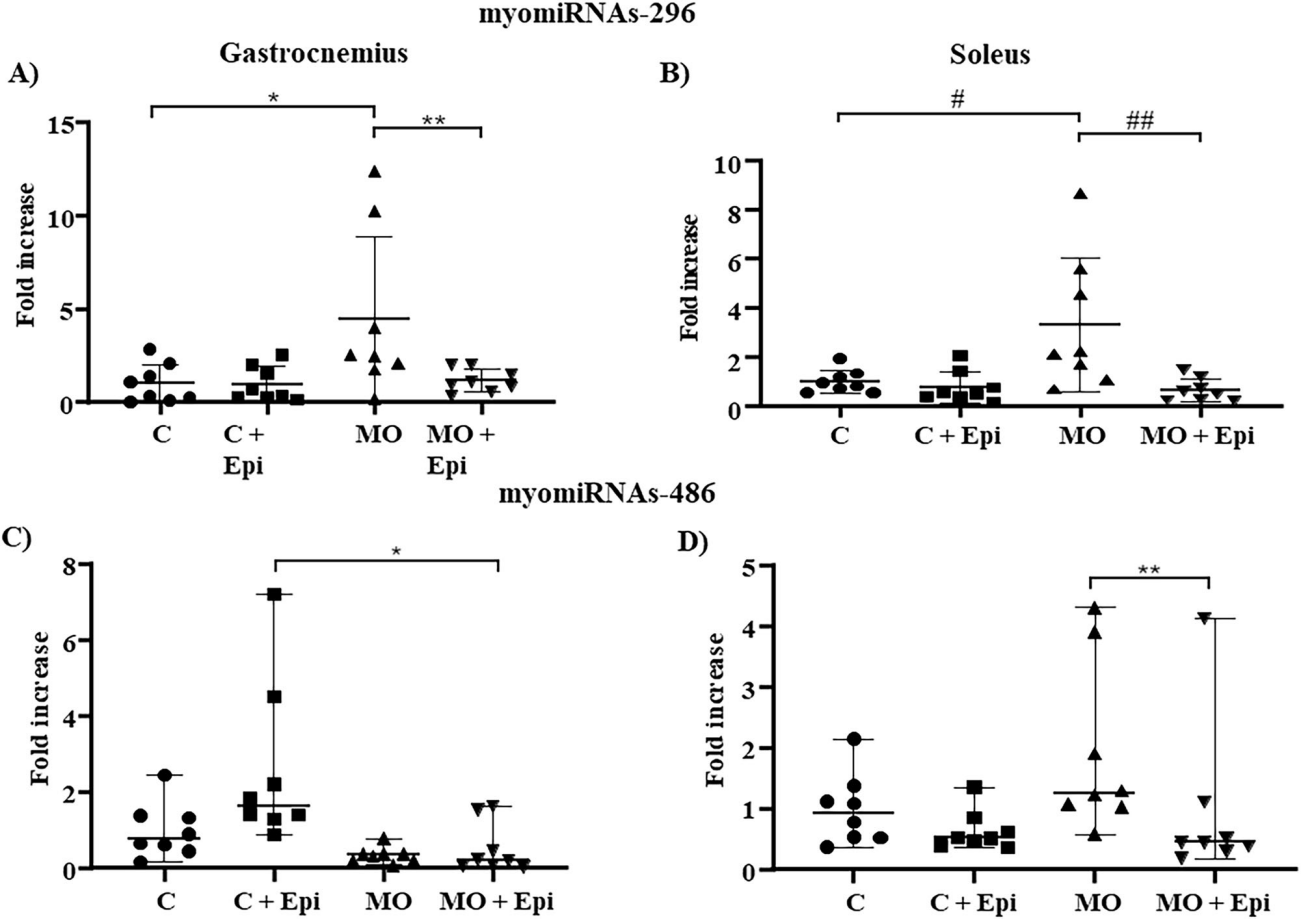
Relative myomiRNA−296 and −486 expression in the gastrocnemius and soleus muscles of the Epi-treated offspring of obese mothers. RT‒qPCR analysis revealed a significant increase in the expression of myomiRNA-296 in the MO gastrocnemius (**Panel A**) (*p = 0.029 vs. C) and MO soleus (**Panel B**) (^#^*p* = **0.019** vs. C). In contrast, epi treatment inhibited this increase (gastrocnemius **p = 0.038 and soleus ^##^ *p* = 0.005). One-way ANOVA and a post hoc Tukey or Bonferroni correction were used to analyze the data for pairwise comparisons. With respect to myomiRNA-486, molecular analysis revealed a significant reduction in expression in the gastrocnemius (**Panel C**) of MO+Epi compared with that in the C+Epi group (*p = 0.008); in the soleus (**Panel D**), Epi cells induced a significant reduction in myomiRNA expression in the MO+Epi group compared with that in the MO group (**p = 0.028). Data are expressed as medians and ranges and were analyzed by the Kruskal‒Wallis nonparametric test followed by a post hoc Dunn test for pairwise comparisons. NS not significant. *n* = 8 rats per group. Epi (-)-epicatechin, C control offspring, MO maternal obesity offspring, C+Epi control offspring treated with Epi, MO+Epi maternal obesity offspring treated with Epi

One-way ANOVA followed by Tukey’s multiple comparisons tests revealed that the MO group presented increased expression of myomiRNA-296 compared with the gastrocnemius C and C+Epi groups (Fig. 4 Panel A, *p* = 0.030 and *p* = 0.027, respectively) and the soleus C group (Fig. 4 Panel B, *p* = 0.016). In both cases, the Epi stimuli prevented this increase in expression (Fig. 4 Panel A, MO vs. MO+Epi, *p* = 0.038; Panel B, MO vs. MO+Epi, *p* = 0.005).

The myomiRNA-486 expression data were evaluated via a nonparametric test, which revealed significant differences between the groups. In the gastrocnemius muscle, miRNA expression was significantly greater in the descendants of control mothers with postnatal Epi treatment (C+Epi) than in the descendants of mothers with obesity and postnatal Epi treatment (MO+Epi) (Fig. 4 Panel C, *p* = 0.008). When the soleus muscle was evaluated, we found that this miRNA expression was significantly lower in the progeny of mothers with obesity and postnatal Epi treatment (MO+Epi) than in the progeny of mothers with obesity but without postnatal Epi treatment (MO) (Fig. 4 Panel D, *p* = 0.028), indicating that the administration of the flavonoid lowers the expression of this miRNA in the context of maternal obesity.

## Discussion

A study by Bayol et al. [31] revealed that an obesogenic environment during pregnancy may reduce the muscle force capacity of offspring. This highlights the importance of fetal programming in muscle development. On the other hand, skeletal muscle-specific miRNAs (named myomiRNAs) play important roles in the development and repair of muscle [12, 32]. In the present study, we analyzed the effects of an obesogenic environment during pregnancy on the expression of some myomiRNAs in the skeletal muscle of offspring, and if treatment with Epi, a flavanol abundant in cacao, modified this effect.

Our results revealed that the obesogenic stimulus did not affect the expression of myomiRNA-31**-**5p; however, the Epi treatment reduced the expression of this myomiRNA in the soleus and gastrocnemius of both the control and obese mothers. In this context, it has been reported that miRNA-31 negatively regulates dystrophin gene expression, and interfering with its activity could help improve dystrophin recovery in Duchenne muscular dystrophy patients, increasing skeletal muscle regeneration [33].

In contrast, our data revealed no change in the expression of myomiRNAs 133 and 136 in the obesogenic environment or with Epi treatment in either the gastrocnemius or soleus. Some data suggest that miRNA-133 participates positively in myoblast proliferation [34]. In addition, the overexpression of miRNA-133a did not affect muscle development, suggesting that it is dispensable [35]. The expression of miRNA-136 5p is reduced in the gastrocnemius as the age of the mice increases [13], indicating that it may be more involved in the embryonic development of the muscle [36].

The intrauterine obesogenic environment significantly increased the expression of myomiRNA-206 in the gastrocnemius, independent of the Epi stimulus. In contrast, in the soleus, the obesogenic environment provoked a reduction in the expression level of this myomiRNA. This myomiRNA is expressed only in skeletal muscle [37], and alterations in its expression have been observed in patients with Duchenne muscular dystrophy [38] and amyotrophic lateral sclerosis [39]. Furthermore, miR-206 is involved in various events in skeletal muscle related to development, regeneration, adaptation, and muscle pathologies [40].

myomiRNA-296 presented a significant increase in expression under intrauterine obesogenic stimulus in the gastrocnemius and soleus. This effect was suppressed when offspring were treated with Epi. A study has shown that myomiRNAs-296-3p was drastically downregulated in the skeletal muscle of pigs with intrauterine growth restriction [13].

However, the overexpression of miRNA-486 may be important in restoring key signaling pathways deregulated in dystrophic muscle that affect muscle growth and function [22]. Our results did not reveal any effect on the regulation of its expression in the gastrocnemius of the offspring exposed to an obesogenic uterine environment or Epi treatment. In contrast, in the offspring soleus, the obesogenic uterine environment induced an increase in the expression of this myomiRNA and a reduction in this effect after Epi treatment. These results suggest that the different effects of environmental factors on distinct muscles could be due to the diverse metabolic and contractile properties of the muscles.

The bioinformatics analysis highlights the potential target genes of myomiRs-31-5p, −133, −136, −206, −296, and −486. Interestingly, most of these genes are involved in biological pathways and molecular functions related to skeletal muscle contraction. Different isoforms of myosin are present in skeletal muscle and influence actin cytoskeleton organization and the contractile properties of muscle fibers [41]. Therefore, myomiRNAs could be important elements in regulating the genes involved in the contraction process of skeletal muscle [42]. This regulation could also participate in the transition of fibers either fast-to-slow or slow-to-fast, depending on physical activity, aging, or pathological conditions, such as hyperthyroidism [41]. Since myomiRNAs play an essential role in muscle development and function, studying their expression could be highly relevant in the search for markers of the progression of diseases such as muscular dystrophies or sarcopenia, in the diagnosis and prognosis of motor neuron diseases or as biomarkers of the efficacy of physical rehabilitation [12]. In addition, obesity affects the quality of skeletal muscle, for example, in sarcopenic obesity [43].

Our study has a limitation, since there are no databases of rat miRNAs and their targets, we had to use a mouse database.

In conclusion, our results show that the analyzed myomiRNAs have individual responses to the intrauterine obesogenic environment and the subsequent postnatal 13 weeks of (-)-epicatechin treatment. However, given the heterogeneity of the animal models used and the variation in data observed, increasing the number of individuals and exploring the effects of Epi treatment over more extended periods would be important in future research. Moreover, in future studies, it would be important to include muscles with different types of myosin and contractile activity, as well as females, to determine whether sex differences exist.

## Supplementary information

Supplementary Fig. 1

Supplementary Table 1

Supplementary Fig. 1

## Data Availability

No datasets were generated or analysed during the current study.
