# Supplementary figures and images for: Effects of Epicatechin on the Expression of MyomiRs−31, −133, −136, −206, −296, and −486 in the Skeletal Muscle of the Offspring of Obese Mothers
Source: Cell Biochem Biophys. 2025 Feb 27;83(3):3177–85. doi: 10.1007/s12013-025-01700-x (PMC12414087; doi:10.1007/s12013-025-01700-x)

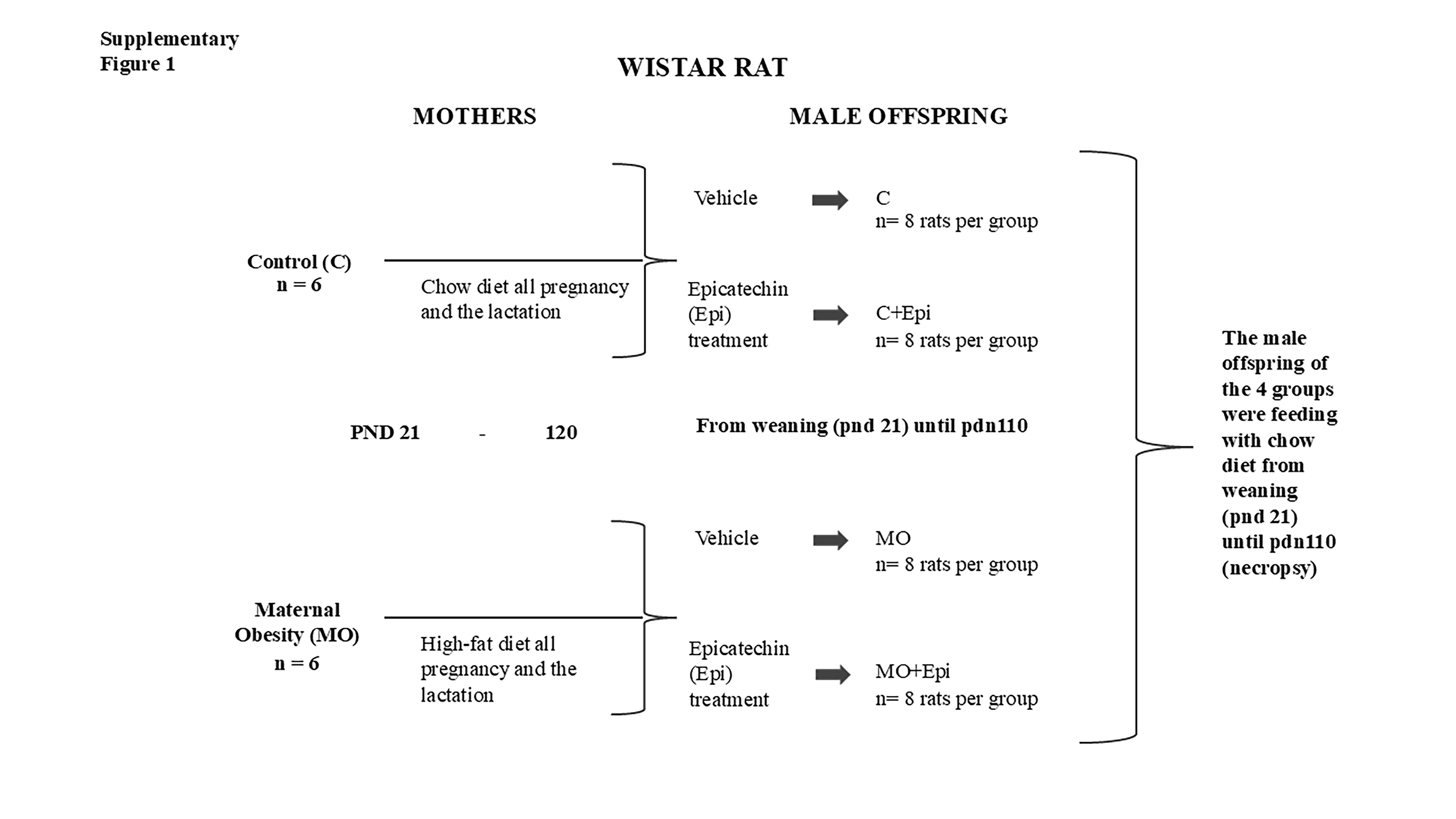

Supplement: Supplementary file 1 — Supplementary Fig. 1 [file 12013_2025_1700_MOESM1_ESM.tif]
