# Supplementary material for: Effects of Epicatechin on the Expression of MyomiRs−31, −133, −136, −206, −296, and −486 in the Skeletal Muscle of the Offspring of Obese Mothers
Source: Cell Biochem Biophys. 2025 Feb 27;83(3):3177–85. doi: 10.1007/s12013-025-01700-x (PMC12414087; doi:10.1007/s12013-025-01700-x)
Supplement: Supplementary file 2 — Supplementary Table 1 [file 12013_2025_1700_MOESM2_ESM.docx]

**Supplementary Table 1. Statistical Analysis of the different myomiRs in Gastrocnemius and Soleus muscles**

| **myomiR-31-5p** |  |  |  |  |  |  |
| --- | --- | --- | --- | --- | --- | --- |
| **Gastrocnemius mucle** |  |  |  |  |  |  |
| **Shapiro-Wilk test** | **C** | **C + Epi long** | **MO** | **MO + Epi long** |  |  |
| W | 0.8411 | 0.9184 | 0.8705 | 0.9239 |  |  |
| *P-*value | 0.0774 | 0.4168 | 0.1523 | 0.462 |  |  |
| Passed normality test (alpha=0.05)? | Yes | Yes | Yes | Yes |  |  |
|  |  |  |  |  |  |  |
| **Two-way ANOVA table** | **SS** | **DF** | **MS** | **F (DFn, DFd)** | **% of total variation** | ***P-*value** |
| Interaction | 0.1293 | 1 | 0.1293 | F (1, 28) = 0.3610 | 0.9743 | 0.5528 |
| Epi treatment | 3.113 | 1 | 3.113 | F (1, 28) = 8.689 | 23.45 | 0.0064 |
| Maternal Diet | 0.000727 | 1 | 0.0007275 | F (1, 28) = 0.00203 | 0.00548 | 0.9644 |
| Residual | 10.03 | 28 | 0.3583 |  |  |  |
|  |  |  |  |  |  |  |
| **Brown-Forsythe test** |  |  |  |  |  |  |
| F (DFn, DFd) | 4.499 (3, 28) |  |  |  |  |  |
| *P-*value | 0.0107 |  |  |  |  |  |
| Are SDs significantly different (P < 0.05)? | Yes |  |  |  |  |  |
|  |  |  |  |  |  |  |
| **Bonferroni's multiple comparisons test** | **Mean Diff.** | **95% CI of diff.** | **Adjusted**  ***P*-value** |  |  |  |
| C vs. C + Epi long | 0.4967 | -0.3530 to 1.346 | 0.6491 |  |  |  |
| C vs. MO | -0.1176 | -0.9673 to 0.7320 | >0.9999 |  |  |  |
| C vs. MO + Epi long | 0.6333 | -0.2163 to 1.483 | 0.2601 |  |  |  |
| C + Epi long vs. MO | -0.6143 | -1.464 to 0.2354 | 0.2975 |  |  |  |
| C + Epi long vs. MO + Epi long | 0.1367 | -0.7130 to 0.9863 | >0.9999 |  |  |  |
| MO vs. MO + Epi long | 0.751 | -0.09869 to 1.601 | 0.109 |  |  |  |

| **Soleus muscle** |  |  |  |  |  |  |
| --- | --- | --- | --- | --- | --- | --- |
|  |  |  |  |  |  |  |
| **Shapiro-Wilk test** | **C** | **C + Epi long** | **MO** | **MO + Epi long** |  |  |
| W | 0.8492 | 0.8695 | 0.9357 | 0.9434 |  |  |
| *P-*value | 0.0935 | 0.1491 | 0.5697 | 0.6446 |  |  |
| Passed normality test (alpha=0.05) | Yes | Yes | Yes | Yes |  |  |
|  |  |  |  |  |  |  |
| **Two-way ANOVA table** | **SS** | **DF** | **MS** | **F (DFn, DFd)** | **% of total variation** | ***P-*value** |
| Interaction | 0.1048 | 1 | 0.1048 | F (1, 28) = 0.4550 | 1.201 | 0.5055 |
| Epi treatment | 1.924 | 1 | 1.924 | F (1, 28) = 8.355 | 22.06 | 0.0074 |
| Maternal Diet | 0.2461 | 1 | 0.2461 | F (1, 28) = 1.069 | 2.821 | 0.3101 |
| Residual | 6.449 | 28 | 0.2303 |  |  |  |
|  |  |  |  |  |  |  |
| **Brown-Forsythe test** |  |  |  |  |  |  |
| F (DFn, DFd) | 2.638 (3, 28) |  |  |  |  |  |
| *P*-value | 0.0691 |  |  |  |  |  |
| Are SDs significantly different (P < 0.05)? | No |  |  |  |  |  |
|  |  |  |  |  |  |  |
| **Tukey's multiple comparisons test** | **Mean Diff.** | **95% CI of diff.** | **Adjusted  *P-*value** |  |  |  |
| C vs. C + Epi long | 0.376 | -0.2792 to 1.031 | 0.4131 |  |  |  |
| C vs. MO | 0.0609 | -0.5942 to 0.7161 | 0.9941 |  |  |  |
| C vs. MO + Epi long | 0.6659 | 0.01071 to 1.321 | 0.0453 |  |  |  |
| C + Epi long vs. MO | -0.315 | -0.9702 to 0.3401 | 0.5627 |  |  |  |
| C + Epi long vs. MO + Epi long | 0.2899 | -0.3653 to 0.9450 | 0.627 |  |  |  |
| MO vs. MO + Epi long | 0.6049 | -0.05025 to 1.260 | 0.0783 |  |  |  |

| **myomiR-133** |  |  |  |  |
| --- | --- | --- | --- | --- |
| **Gastrocnemius muscle** |  |  |  |  |
| **Shapiro-Wilk test** | **C** | **C + Epi long** | **MO** | **MO + Epi long** |
| W | 0.9054 | 0.8423 | 0.8881 | 0.6952 |
| *P*-value | 0.323 | 0.0794 | 0.2248 | 0.002 |
| Passed normality test (alpha=0.05)? | Yes | Yes | Yes | No |
|  |  |  |  |  |
| Kruskal-Wallis test |  |  |  |  |
| *P*-value | 0.9245 |  |  |  |
| Exact or approximate *P*-value? | Approximate |  |  |  |
| *P*-value summary | ns |  |  |  |
| Do the medians vary signif. (P < 0.05)? | No |  |  |  |
| Number of groups | 4 |  |  |  |
| Kruskal-Wallis statistic | 0.4744 |  |  |  |
|  |  |  |  |  |
| **Dunn's multiple comparisons test** | Mean rank diff. | Adjusted P Value |  |  |
| C vs. C + Epi long | -1.125 | >0.9999 |  |  |
| C vs. MO | -2.5 | >0.9999 |  |  |
| C vs. MO + Epi long | -2.875 | >0.9999 |  |  |
| C + Epi long vs. MO | -1.375 | >0.9999 |  |  |
| C + Epi long vs. MO + Epi long | -1.75 | >0.9999 |  |  |
| MO vs. MO + Epi long | -0.375 | >0.9999 |  |  |

| **Soleus muscle** |  |  |  |  |
| --- | --- | --- | --- | --- |
|  |  |  |  |  |
| **Shapiro-Wilk test** | **C** | **C + Epi long** | **MO** | **MO + Epi long** |
| W | 0.6755 | 0.8376 | 0.8824 | 0.9522 |
| *P*-value | 0.0012 | 0.0712 | 0.1986 | 0.7332 |
| Passed normality test (alpha=0.05)? | No | Yes | Yes | Yes |
|  |  |  |  |  |
| **Kruskal-Wallis test** |  |  |  |  |
| P value | 0.4196 |  |  |  |
| Exact or approximate P value? | Approximate |  |  |  |
| *P*-value summary | ns |  |  |  |
| Do the medians vary signif. (P < 0.05)? | No |  |  |  |
| Number of groups | 4 |  |  |  |
| Kruskal-Wallis statistic | 2.824 |  |  |  |
|  |  |  |  |  |
| **Dunn's multiple comparisons test** | **Mean rank diff.** | **Adjusted**  ***P*-value** |  |  |
| C vs. C + Epi long | 3.75 | >0.9999 |  |  |
| C vs. MO | 5 | >0.9999 |  |  |
| C vs. MO + Epi long | 7.75 | 0.5908 |  |  |
| C + Epi long vs. MO | 1.25 | >0.9999 |  |  |
| C + Epi long vs. MO + Epi long | 4 | >0.9999 |  |  |
| MO vs. MO + Epi long | 2.75 | >0.9999 |  |  |

|  |  |  |  |  |
| --- | --- | --- | --- | --- |
| **myomiR-136** |  |  |  |  |
| **Gastrocnemius muscle** |  |  |  |  |
| **Shapiro-Wilk test** | **C** | **C + Epi long** | **MO** | **MO + Epi long** |
| W | 0.837 | 0.8622 | 0.8662 | 0.8222 |
| *P*-value | 0.0702 | 0.1262 | 0.1383 | 0.0492 |
| Passed normality test (alpha=0.05)? | Yes | Yes | Yes | No |
|  |  |  |  |  |
| **Kruskal-Wallis test** |  |  |  |  |
| *P*-value | 0.3825 |  |  |  |
| Exact or approximate P value? | Approximate |  |  |  |
| *P*-value summary | ns |  |  |  |
| Do the medians vary signif. (P < 0.05)? | No |  |  |  |
| Number of groups | 4 |  |  |  |
| Kruskal-Wallis statistic | 3.06 |  |  |  |
|  |  |  |  |  |
| **Dunn's multiple comparisons test** | **Mean rank diff.** | **Adjusted P Value** |  |  |
| C vs. C + Epi long | 6.125 | >0.9999 |  |  |
| C vs. MO | -1.5 | >0.9999 |  |  |
| C vs. MO + Epi long | 0.375 | >0.9999 |  |  |
| C + Epi long vs. MO | -7.625 | 0.6241 |  |  |
| C + Epi long vs. MO + Epi long | -5.75 | >0.9999 |  |  |
| MO vs. MO + Epi long | 1.875 | >0.9999 |  |  |

| **Soleus muscle** |  |  |  |  |
| --- | --- | --- | --- | --- |
|  |  |  |  |  |
| **Shapiro-Wilk test** | **C** | **C + Epi long** | **MO** | **MO + Epi long** |
| W | 0.6682 | 0.7868 | 0.9812 | 0.9051 |
| *P*-value | 0.001 | 0.0206 | 0.9687 | 0.321 |
| Passed normality test (alpha=0.05)? | No | No | Yes | Yes |
|  |  |  |  |  |
| **Kruskal-Wallis test** |  |  |  |  |
| *P*-value | 0.0877 |  |  |  |
| Exact or approximate P value? | Approximate |  |  |  |
| *P*-value summary | ns |  |  |  |
| Do the medians vary signif. (P < 0.05)? | No |  |  |  |
| Number of groups | 4 |  |  |  |
| Kruskal-Wallis statistic | 6.551 |  |  |  |
|  |  |  |  |  |
| **Dunn's multiple comparisons test** | **Mean rank diff.** | **Adjusted**  ***P*-value** |  |  |
| C vs. C + Epi long | 5.75 | >0.9999 |  |  |
| C vs. MO | 0 | >0.9999 |  |  |
| C vs. MO + Epi long | -6.25 | >0.9999 |  |  |
| C + Epi long vs. MO | -5.75 | >0.9999 |  |  |
| C + Epi long vs. MO + Epi long | -12 | 0.0631 |  |  |
| MO vs. MO + Epi long | -6.25 | >0.9999 |  |  |

| **myomiR-206** |  |  |  |  |  |  |
| --- | --- | --- | --- | --- | --- | --- |
| **Gastrocnemius muscle** |  |  |  |  |  |  |
| **Shapiro-Wilk test** | **C** | **C + Epi long** | **MO** | **MO + Epi long** |  |  |
| W | 0.8238 | 0.9546 | 0.9364 | 0.9456 |  |  |
| *P*-value | 0.0511 | 0.7569 | 0.576 | 0.6665 |  |  |
| Passed normality test (alpha=0.05)? | Yes | Yes | Yes | Yes |  |  |
|  |  |  |  |  |  |  |
| **Two-way ANOVA table** | **SS** | **DF** | **MS** | **F (DFn, DFd)** | **% of total variation** | ***P*-value** |
| Interaction | 0.2975 | 1 | 0.2975 | F (1, 28) = 0.3396 | 0.8994 | 0.5647 |
| Epi treatment | 0.03191 | 1 | 0.03191 | F (1, 28) = 0.03642 | 0.0964 | 0.85 |
| Maternal Diet | 8.22 | 1 | 8.22 | F (1, 28) = 9.382 | 24.85 | 0.0048 |
| Residual | 24.53 | 28 | 0.8761 |  |  |  |
|  |  |  |  |  |  |  |
| **Brown-Forsythe test** |  |  |  |  |  |  |
| F (DFn, DFd) | 2.179 (3, 28) |  |  |  |  |  |
| *P*-value | 0.1127 |  |  |  |  |  |
| Are SDs significantly different (P < 0.05)? | No |  |  |  |  |  |
|  |  |  |  |  |  |  |
| **Tukey's multiple comparisons test** | **Mean Diff.** | **95% CI of diff.** | **Adjusted**  ***P*-value** |  |  |  |
| C vs. C + Epi long | 0.256 | -1.022 to 1.534 | 0.9466 |  |  |  |
| C vs. MO | -0.8208 | -2.099 to 0.4570 | 0.3162 |  |  |  |
| C vs. MO + Epi long | -0.9505 | -2.228 to 0.3273 | 0.201 |  |  |  |
| C + Epi long vs. MO | -1.077 | -2.355 to 0.2010 | 0.122 |  |  |  |
| C + Epi long vs. MO + Epi long | -1.206 | -2.484 to 0.07133 | 0.0695 |  |  |  |
| MO vs. MO + Epi long | -0.1297 | -1.407 to 1.148 | 0.9924 |  |  |  |

| **Soleus muscle** |  |  |  |  |  |  |
| --- | --- | --- | --- | --- | --- | --- |
|  |  |  |  |  |  |  |
| **Shapiro-Wilk test** | **C** | **C + Epi long** | **MO** | **MO + Epi long** |  |  |
| W | 0.8359 | 0.8917 | 0.9636 | 0.9414 |  |  |
| *P*-value | 0.0684 | 0.2429 | 0.8435 | 0.6246 |  |  |
| Passed normality test (alpha=0.05)? | Yes | Yes | Yes | Yes |  |  |
|  |  |  |  |  |  |  |
| **Two-way ANOVA table** | **SS** | **DF** | **MS** | **F (DFn, DFd)** | **% of total variation** | ***P*-value** |
| Interaction | 0.06231 | 1 | 0.06231 | F (1, 28) = 0.3477 | 0.8465 | 0.5601 |
| Epi treatment | 0.04299 | 1 | 0.04299 | F (1, 28) = 0.2399 | 0.584 | 0.6281 |
| Maternal Diet | 2.238 | 1 | 2.238 | F (1, 28) = 12.49 | 30.4 | 0.0014 |
| Residual | 5.017 | 28 | 0.1792 |  |  |  |
|  |  |  |  |  |  |  |
| **Brown-Forsythe test** |  |  |  |  |  |  |
| F (DFn, DFd) | 1.186 (3, 28) |  |  |  |  |  |
| *P*-value | 0.3329 |  |  |  |  |  |
| Are SDs significantly different (P < 0.05)? | No |  |  |  |  |  |
|  |  |  |  |  |  |  |
| **Tukey's multiple comparisons test** | **Mean Diff.** | **95% CI of diff.** | **Adjusted *P*-value** |  |  |  |
| C vs. C + Epi long | 0.1616 | -0.4163 to 0.7394 | 0.8702 |  |  |  |
| C vs. MO | 0.6171 | 0.03925 to 1.195 | 0.033 |  |  |  |
| C vs. MO + Epi long | 0.6022 | 0.02431 to 1.180 | 0.0387 |  |  |  |
| C + Epi long vs. MO | 0.4556 | -0.1223 to 1.033 | 0.1616 |  |  |  |
| C + Epi long vs. MO + Epi long | 0.4406 | -0.1372 to 1.019 | 0.1837 |  |  |  |
| MO vs. MO + Epi long | -0.01495 | -0.5928 to 0.5629 | 0.9999 |  |  |  |

| **myomiR-296** |  |  |  |  |  |  |
| --- | --- | --- | --- | --- | --- | --- |
| **Gastrocnemius muscle** |  |  |  |  |  |  |
| **Shapiro-Wilk test** | **C** | **C + Epi long** | **MO** | **MO + Epi long** |  |  |
| W | 0.8831 | 0.836 | 0.8231 | 0.9272 |  |  |
| *P*-value | 0.2015 | 0.0685 | 0.0503 | 0.4911 |  |  |
| Passed normality test (alpha=0.05)? | Yes | Yes | Yes | Yes |  |  |
|  |  |  |  |  |  |  |
| **Two-way ANOVA table** | **SS** | **DF** | **MS** | **F (DFn, DFd)** | **% of total variation** | ***P*-value** |
| Interaction | 21.68 | 1 | 21.68 | F (1, 28) = 3.983 | 9.729 | 0.0558 |
| Epi treatment | 22.5 | 1 | 22.5 | F (1, 28) = 4.135 | 10.1 | 0.0516 |
| Maternal Diet | 26.28 | 1 | 26.28 | F (1, 28) = 4.828 | 11.79 | 0.0364 |
| Residual | 152.4 | 28 | 5.443 |  |  |  |
|  |  |  |  |  |  |  |
| **Brown-Forsythe test** |  |  |  |  |  |  |
| F (DFn, DFd) | 2.462 (3, 28) |  |  |  |  |  |
| *P*-value | 0.0833 |  |  |  |  |  |
| Are SDs significantly different (P < 0.05)? | No |  |  |  |  |  |
|  |  |  |  |  |  |  |
| **Tukey's multiple comparisons test** | **Mean Diff.** | **95% CI of diff.** | **Adjusted**  ***P*-value** |  |  |  |
| C vs. C + Epi long | 0.03095 | -3.154 to 3.216 | >0.9999 |  |  |  |
| C vs. MO | -3.459 | -6.644 to -0.2738 | 0.0294 |  |  |  |
| C vs. MO + Epi long | -0.1352 | -3.320 to 3.050 | 0.9994 |  |  |  |
| C + Epi long vs. MO | -3.49 | -6.675 to -0.3047 | 0.0277 |  |  |  |
| C + Epi long vs. MO + Epi long | -0.1662 | -3.351 to 3.019 | 0.9989 |  |  |  |
| MO vs. MO + Epi long | 3.324 | 0.1386 to 6.508 | 0.0384 |  |  |  |

| **Soleus muscle** |  |  |  |  |  |  |
| --- | --- | --- | --- | --- | --- | --- |
|  |  |  |  |  |  |  |
| **Shapiro-Wilk test** | **C** | **C + Epi long** | **MO** | **MO + Epi long** |  |  |
| W | 0.8969 | 0.8322 | 0.8708 | 0.8716 |  |  |
| *P*-value | 0.2706 | 0.0626 | 0.1535 | 0.1562 |  |  |
| Passed normality test (alpha=0.05)? | Yes | Yes | Yes | Yes |  |  |
|  |  |  |  |  |  |  |
| **Two-way ANOVA table** | **SS** | **DF** | **MS** | **F (DFn, DFd)** | **% of total variation** | ***P*-value** |
| Interaction | 12.06 | 1 | 12.06 | F (1, 28) = 5.770 | 12.4 | 0.0232 |
| Epi treatment | 17.04 | 1 | 17.04 | F (1, 28) = 8.153 | 17.52 | 0.008 |
| Maternal Diet | 9.627 | 1 | 9.627 | F (1, 28) = 4.606 | 9.9 | 0.0407 |
| Residual | 58.52 | 28 | 2.09 |  |  |  |
|  |  |  |  |  |  |  |
| **Brown-Forsythe test** |  |  |  |  |  |  |
| F (DFn, DFd) | 3.779 (3, 28) |  |  |  |  |  |
| *P*-value | 0.0215 |  |  |  |  |  |
| Are SDs significantly different (P < 0.05)? | Yes |  |  |  |  |  |
|  |  |  |  |  |  |  |
| **Bonferroni's multiple comparisons test** | **Mean Diff.** | **95% CI of diff.** | **Adjusted**  ***P*-value** |  |  |  |
| C vs. C + Epi long | 0.2316 | -1.820 to 2.284 | >0.9999 |  |  |  |
| C vs. MO | -2.325 | -4.377 to -0.2727 | 0.0196 |  |  |  |
| C vs. MO + Epi long | 0.3624 | -1.690 to 2.414 | >0.9999 |  |  |  |
| C + Epi long vs. MO | -2.556 | -4.608 to -0.5043 | 0.0086 |  |  |  |
| C + Epi long vs. MO + Epi long | 0.1308 | -1.921 to 2.183 | >0.9999 |  |  |  |
| MO vs. MO + Epi long | 2.687 | 0.6351 to 4.739 | 0.0053 |  |  |  |

| **myomiR-486** |  |  |  |  |
| --- | --- | --- | --- | --- |
| **Gastrocnemius muscle** |  |  |  |  |
| **Shapiro-Wilk test** | **C** | **C + Epi long** | **MO** | **MO + Epi long** |
| W | 0.9079 | 0.7583 | 0.8783 | 0.717 |
| *P*-value | 0.3396 | 0.0101 | 0.1814 | 0.0035 |
| Passed normality test (alpha=0.05)? | Yes | No | Yes | No |
|  |  |  |  |  |
| **Kruskal-Wallis test** |  |  |  |  |
| *P*-value | 0.0018 |  |  |  |
| Exact or approximate P value? | Approximate |  |  |  |
| *P*-value summary | ** |  |  |  |
| Do the medians vary signif. (P < 0.05)? | Yes |  |  |  |
| Number of groups | 4 |  |  |  |
| Kruskal-Wallis statistic | 14.96 |  |  |  |
|  |  |  |  |  |
| **Dunn's multiple comparisons test** | **Mean rank diff.** | **Adjusted**  ***P*-value** |  |  |
| C vs. C + Epi long | -7.625 | 0.6241 |  |  |
| C vs. MO | 8.25 | 0.4716 |  |  |
| C vs. MO + Epi long | 7.375 | 0.6952 |  |  |
| C + Epi long vs. MO | 15.88 | 0.0043 |  |  |
| C + Epi long vs. MO + Epi long | 15 | 0.0083 |  |  |
| MO vs. MO + Epi long | -0.875 | >0.9999 |  |  |

| **Soleus muscle** |  |  |  |  |
| --- | --- | --- | --- | --- |
|  |  |  |  |  |
| **Shapiro-Wilk test** | **C** | **C + Epi long** | **MO** | **MO + Epi long** |
| W | 0.9058 | 0.7858 | 0.7952 | 0.5842 |
| *P*-value | 0.3254 | 0.0201 | 0.0254 | 0.0001 |
| Passed normality test (alpha=0.05)? | Yes | No | No | No |
|  |  |  |  |  |
| **Kruskal-Wallis test** |  |  |  |  |
| *P*-value | 0.0187 |  |  |  |
| Exact or approximate P value? | Approximate |  |  |  |
| *P*-value summary | * |  |  |  |
| Do the medians vary signif. (P < 0.05)? | Yes |  |  |  |
| Number of groups | 4 |  |  |  |
| Kruskal-Wallis statistic | 9.98 |  |  |  |
|  |  |  |  |  |
| **Dunn's multiple comparisons test** | **Mean rank diff.** | **Adjusted**  ***P*-value** |  |  |
| C vs. C + Epi long | 6 | >0.9999 |  |  |
| C vs. MO | -5.625 | >0.9999 |  |  |
| C vs. MO + Epi long | 7.625 | 0.6241 |  |  |
| C + Epi long vs. MO | -11.63 | 0.0792 |  |  |
| C + Epi long vs. MO + Epi long | 1.625 | >0.9999 |  |  |
| MO vs. MO + Epi long | 13.25 | 0.0284 |  |  |
